# Identification of a three-gene expression signature of poor-prognosis breast carcinoma

**DOI:** 10.1186/1476-4598-3-37

**Published:** 2004-12-20

**Authors:** Ivan Bièche, Sengül Tozlu, Igor Girault, Rosette Lidereau

**Affiliations:** 1 Laboratoire d'Oncogénétique – INSERM E0017, Centre René Huguenin, St-Cloud, France; 2 Laboratoire de Génétique Moléculaire – UPRES EA 3618, Faculté des Sciences Pharmaceutiques et Biologiques, Université René Descartes – Paris V, Paris, France

**Keywords:** Breast cancer, Gene expression profiling, Real-time RT-PCR quantification, Prognostic value

## Abstract

**Background:**

The clinical course of breast cancer is difficult to predict on the basis of established clinical and pathological prognostic criteria. Given the genetic complexity of breast carcinomas, it is not surprising that correlations with individual genetic abnormalities have also been disappointing. The use of gene expression profiles could result in more accurate and objective prognostication.

**Results:**

To this end, we used real-time quantitative RT-PCR assays to quantify the mRNA expression of a large panel (n = 47) of genes previously identified as candidate prognostic molecular markers in a series of 100 ERα-positive breast tumor samples from patients with known long-term follow-up. We identified a three-gene expression signature (*BRCA2*, *DNMT3B *and *CCNE1*) as an independent prognostic marker (P = 0.007 by univariate analysis; P = 0.006 by multivariate analysis). This "poor prognosis" signature was then tested on an independent panel of ERα-positive breast tumors from a well-defined cohort of 104 postmenopausal breast cancer patients treated with primary surgery followed by adjuvant tamoxifen alone: although this "poor prognosis" signature was associated with shorter relapse-free survival in univariate analysis (P = 0.029), it did not persist as an independent prognostic factor in multivariate analysis (P = 0.27).

**Conclusion:**

Our results confirm the value of gene expression signatures in predicting the outcome of breast cancer.

## Background

Breast carcinoma is the most common female cancer and is showing an alarming year-on-year increase. Most patients do not die as a result of the primary tumor but from metastatic invasion. The mean 5-year relapse-free survival rate is about 60% overall, but differs significantly between patients with forms that rapidly metastasize and those with less aggressive forms.

Current clinical, pathological and biological parameters, *i.e. *age, menopausal status, lymph-node status, macroscopic tumor size, histological grade and estrogen receptor status, fail to accurately predict clinical behavior.

Breast cancer initiation and progression is a process involving multiple molecular alterations, many of which are reflected by changes in gene expression in malignant cells. Many clinical studies have attempted to identify correlations between altered expression of individual genes and breast cancer outcome, but often with contradictory results. Examples of such genes include *ERBB2*, *CCDN1*, *MYC*, *UPA *and *PAI1 *[1-3]. It is thus likely that these genes have limited predictive power when considered in isolation, but that their clinical relevance may be increased when several genes are considered together.

The recent development of effective tools for monitoring gene expression on a large scale is providing new insights into the involvement of gene networks and regulatory pathways in various tumor processes [4]. It has also led to the discovery of new diagnostic and prognostic indicators, and to the identification of new molecular targets for drug development [5]. These tools include cDNA microarrays, which can be used to explore the expression of thousands of genes at a time, and real-time RT-PCR assays for more accurate and quantitative studies of the expression of a smaller number of selected candidate genes.

In this study, we used real-time quantitative RT-PCR assays to quantify the mRNA expression of 47 candidate prognostic molecular markers in a series of 100 ERα-positive breast tumor samples. We identified a three-gene expression signature (*BRCA2*, *DNMT3B *and *CCNE1*) associated with poor clinical outcome. We then tested this "poor prognosis" signature on an independent panel of ERα-positive breast tumor samples from a well-defined cohort of 104 postmenopausal breast cancer patients treated with primary surgery followed by adjuvant tamoxifen alone with known long-term follow-up.

## Materials and Methods

### Patients and samples

We analyzed samples from two series of women with primary unilateral ERα-positive breast carcinoma. ERα-positive status was determined at both the protein level by the Dextran-coated charcoal method until 1988 and enzymatic immuno-assay thereafter, and at the mRNA level by real-time quantitative RT-PCR assay [6].

The first series consisted of 100 women whose breast tumors were excised at Centre René Huguenin from 1977 to 1987. The patients (mean age 58.1 years, range 34–91) were pre- or post-menopausal (37 and 63 patients, respectively). Sixty patients received adjuvant therapy, consisting of chemotherapy alone in 14 cases, hormone therapy alone in 15 cases, and both treatments in 31 cases. The standard prognostic factors are presented in Table 1. The median follow-up was 9.3 years (range 1.4–16.2 years). Thirty-seven patients relapsed within 10 years after surgery. The first relapse events consisted of local and/or regional recurrences in 11 patients, metastases in 22 patients, and both events in four patients.

**Table 1:** Characteristics of the first series of 100 ERα-positive breast tumor patients, and relation to RFS

		**RFS**	
		
			
	Number of patients	Number of events (%)^a^	*P *value^b^
			
			
*Age*			NS (0.68)
≤50	32	11 (34.3)	
>50	68	26 (38.2)	
			
*SBR histological grade*^c^			NS (0.14)
I	16	3 (18.7)	
II	51	21 (41.1)	
III	26	13 (50.0)	
			
*Lymph node status*			0.042
Node-negative	34	7 (20.5)	
Node-positive	66	30 (45.4)	
			
*Macroscopic tumor size*^d^			NS (0.97)
≤30 mm	69	26 (37.6)	
>30 mm	24	10 (41.6)	

^a^: First relapses (local and/or regional recurrences, and/or metastases).

^b^: Log-rank test. NS, not significant.

^c^: Scarff Bloom Richardson classification. Information available for 93 patients.

^d^: Information available for 93 patients.

The second series consisted of 104 post-menopausal women whose breast tumors were excised at Centre René Huguenin from 1980 to 1994. The patients (mean age 70.9 years, range 54–86) all received post-operative adjuvant hormone therapy consisting of tamoxifen (20 mg daily for 3–5 years) and no other treatment. The standard prognostic factors are reported in Table 2. The median follow-up was 5.9 years (range 1.4–18.1 years). Thirty-one patients relapsed within 10 years after surgery. The first relapse events consisted of local and/or regional recurrences in five patients, metastases in 24 patients, and both events in two patients.

**Table 2:** Characteristics of the second series of 104 ERα-positive postmenopausal breast tumor patients, and relation to RFS

		**RFS**	
		
			
	Number of patients	Number of events (%)^a^	*P *value^b^
			
			
*Age*			NS (0.92)
≤70	52	17 (32.6)	
>70	52	14 (26.9)	
			
*SBR histological grade*^c^			0.0005
I	13	0	
II	67	17 (25.3)	
III	23	13 (56.5)	
			
*Lymph node status*			NS (0.17)
Node-negative	17	2 (11.7)	
Node-positive	87	29 (33.3)	
			
*Macroscopic tumor size*^d^			0.015
≤30 mm	71	16 (22.5)	
>30 mm	31	14 (45.1)	

^a^: First relapses (local and/or regional recurrences, and/or metastases)

^b^: Log-rank test. NS, not significant.

^c^: Scarff Bloom Richardson classification. Information available for 103 patients.

^d^: Information available for 102 patients.

Complete clinical, histological and biological information was available for the two series of breast cancer patients; no radiotherapy or chemotherapy was given before surgery, and full follow-up took place at Centre René Huguenin. The histological type of the tumor and the number of positive axillary nodes were established at the time of surgery. The malignancy of infiltrating carcinomas was scored according to Scarff Bloom and Richardson's (SBR) histoprognostic system.

Both series of tumor samples were placed in liquid nitrogen until total RNA extraction immediately following surgery.

### Real-time RT-PCR

#### (1) Theoretical basis

Quantitative values are obtained from the cycle number (Ct value) at which the increase in fluorescent signal associated with an exponential growth of PCR products starts to be detected by the laser detector of the ABI Prism 7700 Sequence Detection System (Perkin-Elmer Applied Biosystems, Foster City, CA) using the PE Biosystems analysis software according to the manufacturer's manuals.

The precise amount of total RNA added to each reaction (based on optical density) and its quality (i.e. lack of extensive degradation) are both difficult to assess. We therefore also quantified transcripts of the gene *TBP *(Genbank accession NM_003194) encoding for the TATA box-binding protein (a component of the DNA-binding protein complex TFIID) as an endogeneous RNA control, and normalized each sample on the basis of its *TBP *content.

Results, expressed as N-fold differences in target gene expression relative to the *TBP *gene, termed "N*target*", were determined by the formula: N*target *= 2^ΔCt *sample*^, where ΔCt value of the sample was determined by subtracting the average Ct value of the target gene from the average Ct value of the *TBP *gene.

The N*target *values of the samples were subsequently normalized such that the N*target *value of the tumor sample which contained the smallest amount of target gene mRNA in each tumor series would equal a value of 1.

#### (2) Primers and probes

Primers and probes for *TBP *and the 47 target genes were chosen with the assistance of the computer programs Oligo 5.0 (National Biosciences, Plymouth, MN). We conducted searches in dbEST, htgs and nr databases to confirm the total gene specificity of the nucleotide sequences chosen for the primers and probes, and the absence of single nucleotide polymorphisms. In particular, the primer pairs were selected to be unique when compared with the sequences of the closely related family member genes or of corresponding retropseudogenes. To avoid amplification of contaminating genomic DNA, one of the two primers or the probe was placed at the junction between two exons. Agarose gel electrophoresis allowed us to verify the specificity of PCR amplicons. The list of the 47 target genes tested in this study is indicated in Table 3.

**Table 3:** List of the 47 target genes selected

**Genes^a^**	***Genbank accession number***	**Chromosomal location**	**Description**
*AR*	NM_000044	Xq11.2-q12	Androgen receptor
*AREG*	NM_001657	4q13-q21	Amphiregulin
*ARHC/RhoC*	NM_175744	1p13.1	Ras homolog gene family, member C
*BCL2*	NM_000633	18q21.3	B-cell CLL/lymphoma 2
*BRCA1*	NM_007294	17q21	Breast cancer 1, early onset
*BRCA2*	NM_000059	13q12.3	Breast cancer 2, early onset
*CAV1*	NM_001753	7q31.1	Caveolin 1
*CCND1*	NM_053056	11q13	Cyclin D1
*CCNE1*	NM_001238	19q12	Cyclin E1
*CD44*	NM_000610	11p13	CD44 antigen
*CDH1*	NM_004360	16q22.1	Cadherin 1 (E-cadherin)
*CGA*	NM_000735	6q12-q21	Glycoprotein hormones, alpha polypeptide
*CGB*	NM_000737	19q13.32	Chorionic gonadotropin, beta polypeptide
*CP/Ceruloplasmin*	NM_000096	3q23-q25	Ceruloplasmin
*CXCL12*	NM_000609	10q11.1	Chemokine (C-X-C motif) ligand 12
*CXCR4*	NM_003467	2q21	Chemokine (C-X-C motif) receptor 4
*DNMT3B*	NM_006892	20q11.2	DNA (cytosine-5-)-methyltransferase 3 beta
*EGFR/ERBB1*	NM_005228	7p12	Epidermal growth factor receptor
*ERBB2*	NM_004448	17q21.1	ErbB2
*ERBB3*	NM_001982	12q13	ErbB3
*ERBB4*	NM_005235	2q33.3-q34	ErbB4
*ESR1/ERα*	NM_000125	6q25.1	Estrogen receptor 1 (alpha)
*ESR2/ERβ*	NM_001437	14q	Estrogen receptor 2 (beta)
*ETV4/PEA3/E1AF*	NM_001986	17q21	Ets variant gene 4
*HAS2*	NM-005328	8q24.12	Hyaluronan synthase 2
*HMMR/RHAMM*	NM_012484	5q33.2-qter	Hyaluronan-mediated mobility receptor
*KRT19*	NM_002276	17q21.2	Keratin 19
*MKI67*	NM_002417	10q25-qter	Antigen identified by monoclonal antibody Ki-67
*MYC*	NM_002467	8q24.12-q24.13	c-myc oncogene
*p14/ARF*	NM_058195	9p21	Alternative reading frame p14 (p14ARF)
*p15/CDKN2B*	NM_004936	9p21	Cyclin-dependent kinase inhibitor 2B (p15 CDK inhibitor)
*p16/CDKN2A*	NM_000077	9p21	Cyclin-dependent kinase inhibitor 2A (p16 CDK inhibitor)
*PGR/PR*	NM_000926	11q22-q23	Progesterone receptor
*PLAU/UPA*	NM_002658	10q24	Plasminogen activator, urokinase
*PTGS2/COX2*	NM_000963	1q25.2-q25.3	Prostaglandin-endoperoxide synthase 2
*PTTG1/Securin*	NM_004219	5q35.1	Pituitary tumor-transforming 1
*RB1*	NM_000321	13q14.2	Retinoblastoma 1
*SERPINB2/PAI2*	NM_002575	18q21.3	Plasminogen activator inhibitor type 2
*SERPINB5/Maspin*	NM_002639	18q21.3	Maspin
*SERPINE1/PAI1*	NM_000602	7q21.3-q22	Plasminogen activator inhibitor type 1
*SPP1/Osteopontin*	NM_000582	4q21-q25	Secreted phosphoprotein 1
*SRC*	NM_005417	20q12-q13	c-src oncogene
*TERT*	NM_003219	5p15.33	Telomerase reverse transcriptase
*TFF1/pS2*	NM_003225	21q22.3	Trefoil factor 1
*TIAM1*	NM_003253	21q22.11	T-cell lymphoma invasion and metastasis 1
*TOP2A*	NM_001067	17q21-q22	Topoisomerase (DNA) II alpha 170 kDa
*XLKD1/LYVE-1*	NM_006691	11p15	Extracellular link domain containing 1

^a^LocusLink symbol

#### (3) RNA extraction

Total RNA was extracted from frozen tumor samples by using the acid-phenol guanidinium method. The quality of the RNA samples was determined by electrophoresis through agarose gels and staining with ethidium bromide, and the 18S and 28S RNA bands were visualized under ultraviolet light.

#### (4) cDNA Synthesis

Reverse transcription of total RNA was done in a final volume of 20 μL containing 1X RT buffer (500 μM each dNTP, 3 mM MgCl2, 75 mM KCl, 50 mM Tris-HCl pH 8.3), 20 units of RNasin RNase inhibitor (Promega, Madison, WI), 10 mM DDT, 100 units of Superscript II RNase H- reverse transcriptase (Invitrogen, Cergy Pontoise, France), 3 μM random hexamers (Pharmacia, Uppsala, Sweden) and 1 μg of total RNA. The samples were incubated at 20°C for 10 min and 42°C for 30 min, and reverse transcriptase was inactivated by heating at 99°C for 5 min and cooling at 5°C for 5 min.

#### (5) PCR amplification

All PCR reactions were performed using a ABI Prism 7700 Sequence Detection System (Perkin-Elmer Applied Biosystems). PCR was performed using either the TaqMan^® ^PCR Core Reagents kit or the SYBR^® ^Green PCR Core Reagents kit (Perkin-Elmer Applied Biosystems). The thermal cycling conditions comprised an initial denaturation step at 95°C for 10 min and 50 cycles at 95°C for 15 s and 65°C for 1 min.

### Statistical Analysis

The distributions of the gene mRNA levels were characterized by their median values and ranges. Relationships between mRNA levels of the different target genes and comparison between the target gene mRNA levels and the clinical parameters were estimated using nonparametric tests: the Mann-Whitney *U *test (link between 1 qualitative parameter and 1 quantitative parameter) and the Spearman rank correlation test (link between 2 quantitative parameters). Differences between the two populations were judged significant at confidence levels greater than 95% (p < 0.05).

To visualize the efficacy of a molecular marker to discriminate two populations (in the absence of an arbitrary cutoff value), we summarized the data in a ROC (receiver operating characteristic) curve [7]. This curve plots the sensibility (true positives) on the *Y *axis against 1 – the specificity (false positives) on the *X *axis, considering each value as a possible cutoff value. The AUC (area under curves) was calculated as a single measure for the discriminate efficacy of a molecular marker. When a molecular marker has no discriminative value, the ROC curve will lie close to the diagonal and the AUC is close to 0.5. When a test has strong discriminative value, the ROC curve will move up to the upper left-hand corner (or to the lower right-hand corner) and the AUC will be close to 1.0 (or 0).

Hierarchical clustering was performed using the GenANOVA software [8].

Relapse-free survival (RFS) was determined as the interval between diagnosis and detection of the first relapse (local and/or regional recurrences, and/or metastases).

Survival distributions were estimated by the Kaplan-Meier method [9], and the significance of differences between survival rates was ascertained using the log-rank test [10]. Cox's proportional hazards regression model [11] was used to assess prognostic significance.

## Results

### mRNA expression of 47 genes in 100 ERα-positive breast tumors

The results for the 47 genes are summarized in table 4, with medians and ranges of mRNA levels in patients who relapsed (n = 37) and those who did not (n = 63).

**Table 4:** Relationships between the prognostic (+/- relapses) and the mRNA levels of the 47 selected genes in 100 ERα-positive breast tumors

**GENES**	**Tumors without relapses (n = 63)**	**Tumors with relapses (n = 37)**	***P*^a^**	**ROC-AUC^b^**
***BRCA2***	**4.6 (1.0–12.4)^c^**	**7.1 (1.9–18.8)**	**0.0011**	**0.696 (0.59–0.80)^d^**
***DNMT3B***	**3.0 (1.0–13.6)**	**4.6 (1.2–17.4)**	**0.0015**	**0.690 (0.58–0.80)**
***CCNE1***	**6.2 (1.0–36.9)**	**8.9 (3.1–82.5)**	**0.0038**	**0.674 (0.57–0.78)**
***HMMR/RHAMM***	**18.9 (1.0–163.5)**	**30.1 (3.8–186.5)**	**0.0068**	**0.663 (0.55–0.77)**
***MKI67***	**9.1 (1.0–49.8)**	**14.4 (1.7–54.9)**	**0.016**	**0.645 (0.53–0.75)**
***TERT***	**18.7 (1.0–121.9)**	**22.1 (1.8–135.8)**	**0.049**	**0.618 (0.50–0.73)**
*TOP2A*	40.9 (1.0–306)	55.6 (6.1–317)	NS	0.605 (0.49–0.72)
*PLAU/UPA*	4.6 (1.0–36.4)	5.8 (1.4–34.0)	NS	0.588 (0.47–0.70)
*CGB*	4.2 (1.0–31.2)	6.4 (1.4–32.8)	NS	0.579 (0.46–0.70)
*ERBB2*	14.0 (1.0–175)	16.3 (4.2–179.8)	NS	0.573 (0.46–0.69)
*BRCA1*	11.9 (1.0–44.5)	14.3 (1.8–62.5)	NS	0.569 (0.45–0.69)
*CXCR4*	6.5 (1.0–40.5)	7.5 (1.5–71.5)	NS	0.569 (0.45–0.68)
*PTTG1/Securin*	1.9 (1.0–26.9)	1.9 (1.2–33.1)	NS	0.566 (0.45–0.68)
*SRC*	2.6 (1.0–4.3)	2.9 (1.4–10.2)	NS	0.561 (0.45–0.67)
*p16/CDKN2A*	3.4 (1.0–107.4)	4.4 (1.1–136.7)	NS	0.560 (0.44–0.68)
*AREG*	89.3 (1.0–5667)	110.1 (3.1–3301)	NS	0.555 (0.44–0.67)
*SERPINE1/PAI1*	3.8 (1.0–21.4)	4.5 (1.3–21.8)	NS	0.554 (0.44–0.67)
*ERBB3*	2.6 (1.0–10.7)	3.3 (1.2–13.4)	NS	0.552 (0.44–0.67)
*SERPINB5/Maspin*	12.6 (1.0–321)	16.4 (1.0–718)	NS	0.551 (0.43–0.67)
*CDH1*	11.3 (1.0–32.6)	13.9 (1.5–33.3)	NS	0.549 (0.43–0.67)
*p15/CDKN2B*	3.5 (1.0–16.2)	4.2 (1.0–34.9)	NS	0.548 (0.43–0.67)
*SPP1/Osteopontin*	43.3 (1.0–1403)	56.8 (2.1–941)	NS	0.548 (0.42–0.68)
*ETV4/PEA3/E1AF*	5.1 (1.0–49.3)	6.9 (1.8–62.0)	NS	0.545 (0.43–0.66)
*CP/Ceruloplasmin*	33.5 (1.0–9815)	81.5 (1.0–33943)	NS	0.545 (0.42–0.67)
*SERPINB2/PAI2*	13.0 (1.0–498)	15.3 (1.0–1652)	NS	0.535 (0.42–0.65)
*TIAM1*	13.6 (1.0–55.9)	13.3 (3.9–83.2)	NS	0.526 (0.41–0.64)
*RB1*	4.2 (1.0–7.4)	4.3 (1.5–7.7)	NS	0.520 (0.40–0.64)
*AR*	54.2 (1.0–219)	64.8 (1.0–211)	NS	0.518 (0.40–0.64)
*HAS2*	6.5 (1.0–40.8)	6.4 (1.4–31.9)	NS	0.516 (0.40–0.63)
*TFF1/pS2*	1772 (1.0–138 545)	1783 (3–55 878)	NS	0.509 (0.39–0.62)
*ESR2/ERβ*	28.2 (1.0–368)	25.3 (1.4–219)	NS	0.500 (0.38–0.62)
*ERBB4*	141 (1.0–1489)	143 (2.1–1062)	NS	0.483 (0.37–0.60)
*KRT19*	14.4 (1.6–99.1)	10.8 (1.0–57.1)	NS	0.482 (0.36–0.60)
*ESR1/ERα*	25.5 (1.0–508)	21.7 (1.2–498)	NS	0.479 (0.36–0.60)
*CXCL12*	12.1 (1.3–36.1)	9.6 (1.0–30.5)	NS	0.464 (0.35–0.58)
*MYC*	8.1 (1.0–35.5)	7.5 (1.0–51.2)	NS	0.464 (0.35–0.58)
*EGFR/ERBB1*	8.3 (1.2–108)	6.2 (1.0–66.8)	NS	0.462 (0.34–0.58)
*ARHC/RhoC*	6.9 (1.0–192)	6.3 (1.0–17.2)	NS	0.458 (0.34–0.58)
*p14/ARF*	4.9 (1.4–68.1)	4.4 (1.0–61.2)	NS	0.457 (0.34–0.57)
*XLKD1/LYVE-1*	4.5 (1.4–10.9)	3.7 (1.0–10.7)	NS	0.448 (0.33–0.57)
*CD44*	3.1 (1.2–9.6)	2.7 (1.0–8.4)	NS	0.440 (0.32–0.56)
*CGA*	17.6 (1.0–16 552)	6.4 (1.0–5 836)	NS	0.425 (0.31–0.54)
*CAV1*	7.4 (1.1–30.7)	5.6 (1.0–26.6)	NS	0.422 (0.31–0.54)
*BCL2*	4.9 (1.2–13.3)	3.2 (1.0–11.8)	NS	0.415 (0.30–0.53)
*PGR/PR*	277 (1.0–8 034)	97 (1.0–4 551)	NS	0.412 (0.29–0.53)
*PTGS2/COX2*	4.6 (1.0–154)	3.0 (1.0–14.8)	NS	0.397 (0.28–0.51)
***CCND1***	**6.3 (1.2–45.3)**	**4.0 (1.0–21.3)**	**0.042**	**0.378 (0.26–0.50)**

^a:^*P *value, Mann-Whitney *U *test ; NS, not significant

^b:^ROC (*Receiver Operating Characteristics*) – AUC (*Area Under Curve*) analysis

^c:^Median (range) of gene mRNA levels

^d:^AUC value (95% confidence interval)

Seven genes showed significantly different expression according to relapse status (P < 0.05), namely *BRCA2*, *DNMT3B*, *CCNE1*, *HMMR/RHAMM*, *MKI67*, *TERT *and *CCND1*. The prognostic performance of these 7 genes was also assessed using ROC-AUC analysis. *BRCA2 *emerged as the most discriminatory marker of relapse status (ROC-AUC, 0.696). The mRNA expression of this gene, as well as *DNMT3B*, *CCNE1*, *HMMR/RHAMM*, *MKI67 *and *TERT*, was higher in patients who relapsed than in patients who did not relapse, while only *CCND1 *mRNA expression was lower in patients who relapsed.

**Figure 1:**
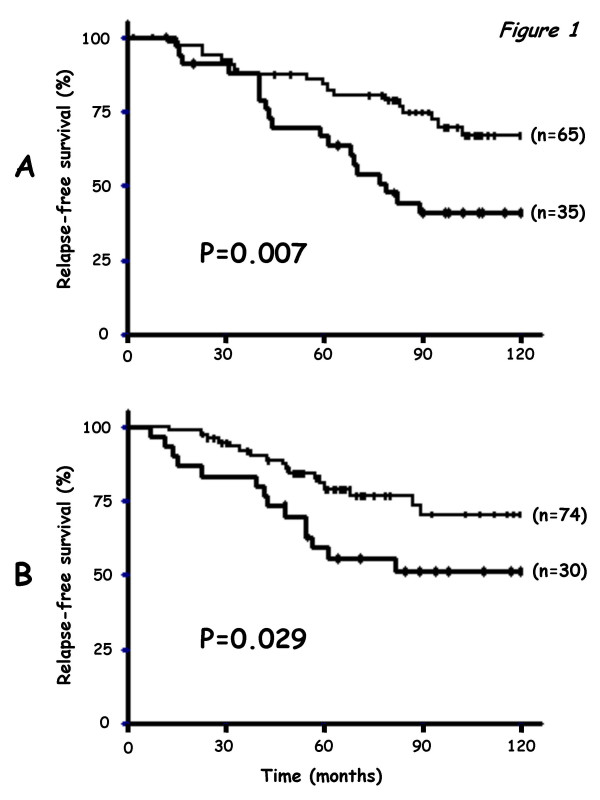
Relationship between RFS and the three-gene expression signature in the initial series of 100 ERα-positive breast tumor samples (A) and in an independent series of 104 ERα-positive postmenopausal breast tumor samples (B).

On hierarchically clustering the tumor samples according to the expression of the three most discriminatory genes *i.e. *the genes with the highest ROC-AUC values (*BRCA2*, *DNMT3B *and *CCNE1*, ROC-AUC: 0.696, 0.690 and 0.674, respectively), the patient population fell into two subgroups (65 and 35 subjects, respectively) with significantly different relapse-free survival curves (log-rank test, P = 0.007; Figure 1A) (5-year RFS rate 66.9% ± 8.1 versus 83.9% ± 4.6; 10-year RFS rate 41.0% ± 8.7 versus 67.0% ± 6.6).

The prognostic value of a two-gene expression signature based on only *BRCA2 *and *DNMT3B *was lower than that of the three-gene expression signature. The addition of *HMMR/RHAMM *and/or *MKI67 *to the three-gene signature provided no additional prognostic value.

Using a Cox proportional hazards model, we also assessed the prognostic value, for RFS, of parameters that were significant or near-significant (P < 0.2) in univariate analysis, *i.e. *SBR grade, lymph-node status (Table 1) and the three-gene expression signature (Figure 1A). Only the prognostic significance of the three-gene expression signature persisted [P = 0.006; regression coefficient = 0.86; relative risk (95% confidence interval) = 2.37 (1.27–4.43)]. The prognostic significance of these three parameters for RFS, calculated in terms of the relative risk, did not change after adjustment for age and macroscopic tumor size (data not shown).

### Validation of the three-gene expression signature in an independent series of 104 ERα-positive postmenopausal breast tumor samples

The results for each of the three genes are summarized in table 5, with medians and ranges of mRNA levels in the 31 patients who relapsed and the 73 patients who did not relapse, as well as ROC-AUC values. As in the initial tumor series, *BRCA2*, *DNMT3B *and *CCNE1 *mRNA levels were significantly higher in patients who relapsed than in those who did not relapse.

**Table 5:** Relationships between the prognostic (+/- relapses) and the mRNA levels of *BRCA2*, *DNMT3B *and *CCNE1 *in 104 ERα-positive postmenopausal breast tumors

**GENES**	**Tumors without relapses (n = 73)**	**Tumors with relapses (n = 31)**	***P*^a^**	**ROC-AUC^b^**
*BRCA2*	4.7 (1.0–23.6^c^	7.5 (1.4–38.4)	0.0018	0.694 (0.58–0.80)^d^
*DNMT3B*	3.6 (1.0–27.8)	6.2 (2.3–74.1)	0.00052	0.716 (0.61–0.82)
*CCNE1*	6.4 (1.0–46.2)	9.0 (1.3–62.8)	0.028	0.636 (0.51–0.76)

^a:^*P *value, Mann-Whitney *U *test.

^b:^ROC (*Receiver Operating Characteristics*) – AUC (*Area Under Curve*) analysis

^c:^Median (range) of gene mRNA levels

^d:^AUC value (95% confidence interval)

On hierarchical clustering of the samples, the three-gene expression signature dichotomized the 104 patients into two subgroups (n = 30 and n = 74, respectively) of similar sizes to those of the initial patient population (n = 35 and n = 65, respectively).

The "poor prognosis" signature was again associated with shorter relapse-free survival in this independent tumor series (log-rank test, P = 0.029; Figure 1B) (5-year RFS 59.2% ± 9.1 versus 80.7% ± 4.8; 10-year RFS 51.2% ± 9.50 versus 70.4% ± 6.5).

Multivariate analysis based on a Cox proportional hazards model showed that, among the parameters that were significant or near-significant (P < 0.2) in univariate analysis, *i.e. *SBR grade, lymph-node status, macroscopic tumor size (Table 2) and the three-gene expression signature (Figure 1B), only SBR grade was an independent predictor of RFS (P = 0.00023); the three-gene expression signature only showed a trend towards significance (P = 0.27).

## Discussion

We used real-time quantitative RT-PCR assays to quantify the mRNA expression of 47 genes previously identified as candidate prognostic molecular markers in 100 ERα-positive breast tumor samples. We identified a three-gene expression signature (*BRCA2*, *DNMT3B *and *CCNE1*) with independent prognostic significance in breast cancer (P = 0.007 by univariate analysis; P = 0.006 by multivariate analysis). This "poor prognosis" signature was then tested on an independent set of 104 ERα-positive breast tumors from a well-defined cohort of postmenopausal breast cancer patients treated with primary surgery followed by adjuvant tamoxifen alone. It was found to be significant in univariate analysis (P = 0.029), but not in multivariate analysis (P = 0.27). We have previously published individual data for 18 of these 47 genes, namely *ERBB1-4 *[12]; *MYC *[13]; *TERT *[14]; *CCND1 *[15]; *CGB*, *CGA*, *ERα*, *ERβ*, *PR*, *PS2 *[16]; *AR *[17]; *DNMT3B *[18], *PAI1*, *PAI2 *and *UPA *[19], obtained using the same real-time RT-PCR method but in a heterogeneous series of 130 ERα-positive and ERα-negative breast tumors.

Large-scale real-time quantitative RT-PCR is a promising complement and/or alternative to cDNA microarrays for molecular tumor profiling. CDNA microarrays have been used to identify gene expression profiles associated with poor outcome in breast cancer [20-26], but discrepancies have been reported. For example, only 2 of 456 genes identified by Sorlie *et al. *[21] was among the 70 genes identified by van de Vijver *et al. *[24].

These discrepancies may be due to the clinical, histological and ethnic heterogeneity of breast cancer, but also to the fact that breast tumors consist of many different cell types – not just tumoral epithelial cells, but also additional epithelial cell types, stromal cells, endothelial cells, adipose cells, and infiltrating lymphocytes. Real-time RT-PCR requires smaller starting amounts of total RNA (about 1–2 ng per target gene) than do cDNA microarrays, making it more suitable for analyzing small tumor samples, cytopuncture specimens and microdissected samples. Real-time RT-PCR also has a linear dynamic range of at least four orders of magnitude, meaning that samples do not need to contain equal starting amounts of RNA. Real-time RT-PCR is also more suitable than cDNA microarrays for analyzing weak variations in gene expression and weakly expressed genes (e.g. *TERT *as in the present study), and for distinguishing among closely related family member genes or alternatively spliced specific transcripts (e.g. the gene cluster *p14/ARF*, *p16/CDKN2A *and *p15/CDKN2B *as in the present study). Finally, real-time quantitative RT-PCR assay is a reference in terms of its performance, accuracy, sensitivity and throughput for nucleic acid quantification, and is more appropriate for routine use in clinical laboratories, being simple, rapid and yielding good inter-laboratory agreement and statistical confidence values.

In this study, we chose to include well known genes involved in breast carcinogenesis reported in the literature and representing a broad range of cellular functions, such as cell cycle control, cell-cell interactions, signal transduction pathways, apoptosis and angiogenesis (Table 3). Many important genes were not studied, but our results nevertheless demonstrate the usefulness of real time RT-PCR by identifying a potentially useful gene expression signature with prognostic significance.

The comparison of median target gene mRNA levels between patients who did and did not relapse provided two interesting results: (a) *ERBB2 *mRNA levels were very similar between the two subgroups, with ROC-AUC values close to 0.5 (ROC-AUC, 0.573), confirming that the *ERBB2 *mRNA expression level is not a major prognostic factor in breast cancer; (b) *ESR1/ERα *mRNA levels were not different between the two subgroups (ROC-AUC, 0.530), suggesting that the ESR1/ERα mRNA expression level in ERα-positive tumors is not predictive of outcome.

The three-gene expression signature predictive of subsequent relapse status comprised genes involved in cell cycle control (*CCNE1*), DNA methylation (*DNMT3B*) and DNA damage repair (*BRCA2*). This gene expression signature is an interesting candidate for routine clinical use, especially as the three genes encode well-characterized proteins for which specific antibodies are already commercially available. Furthermore, the three protein products are amenable to pharmacological control.

*CCNE1 *codes for cyclin E, a protein involved in regulating the early G1 to late G1 phase "restriction point traversal", an irreversible commitment to undergo one cell division [27]. We found that high *CCNE1 *mRNA levels were associated with poor outcome, confirming published data suggesting that cyclin E upregulation may be a major prognostic marker in breast cancer [28-31].

*BRCA2 *codes for a ubiquitously expressed tumor suppressor protein involved in processes fundamental to all cells, including DNA repair, DNA recombination and cell cycle checkpoint control [32]. We found that high *BRCA2 *mRNA levels were associated with poor outcome and correlated positively and strongly with cell proliferation. By hierarchical clustering analysis of the 47 genes, we identified *BRCA2 *as the leading gene in a cluster of proliferation genes also including *TERT*, *BRCA1*, *HMMR/RHAMM *and *MKI67 *(data not shown). We also observed a strong positive link between *BRCA2 *and *MKI67*, which encodes the proliferation-related Ki-67 antigen (Spearman rank correlation test: r=+0.670, P < 10^-7^). The observed strong associations between *BRCA2*, *HMMR/RHAMM *and *MKI67 *mRNA expression explain why four- and five-gene expression signatures, comprising *HMMR/RHAMM *alone or together with *MKI67*, showed no additional prognostic value relative to the three-gene signature.

Our results for *BRCA2 *expression *ex vivo *are in keeping with reports from several authors [33,34] showing that *BRCA2 *mRNA expression is upregulated in rapidly proliferating cells *in vitro*. Our results are also in agreement with those of Egawa *et al*. [35] showing that high *BRCA2 *expression carries a poor prognosis in breast cancer. This link between *BRCA2 *overexpression and poor outcome should be taken into account when evaluating future *BRCA2*-based therapeutic approaches to breast cancer.

Finally, *DNMT3B*, the third gene in our expression signature, codes for one of the three functional DNA methyltransferases (*DNMT1*, *DNMT3A *and *DNMT3B*) that catalyze the transfer of methyl groups to the 5-position of cytosine (DNA methylation). We previously showed that, among the three DNA methyltransferases (*DNMT1*, *DNMT3A *and *DNMT3B*), only *DNMT3B *overexpression is associated with poor outcome in breast cancer [18]. *DNMT3B *(like *DNMT3A*) is known to be a *de novo *methylator of CpG sites. Abnormal DNA methylation is thought to be a major early event in the development of tumors characterized by widespread genome hypomethylation leading to chromosome instability and localized DNA hypermethylation; the latter may be important in tumorigenesis by silencing tumor suppressor genes [36].

## Conclusions

In conclusion, by studying the expression of 47 genes previously identified as candidate prognostic markers in breast cancer, we identified a three-gene expression signature (*BRCA2*, *DNMT3B *and *CCNE1*) with prognostic significance. The practical value of this signature remains to be validated in large prospective randomized studies.

## Abbreviations

*ERα*, estrogen receptor alpha; RT-PCR, reverse transcriptase-polymerase chain reaction.

## Authors' contributions

Real-time RT-PCR have been carried out by ST and IG. IB and RL interpreted the result, performed bioinformatics and statistical analyses.
